# Resolving ambiguity in the phylogenetic relationship of genotypes A, B, and C of hepatitis B virus

**DOI:** 10.1186/1471-2148-13-120

**Published:** 2013-06-11

**Authors:** Yueming Jiang, Minxian Wang, Hongxiang Zheng, Wei R Wang, Li Jin, Yungang He

**Affiliations:** 1 State Key Laboratory of Genetic Engineering, School of Life Sciences and Institutes of Biomedical Sciences, Fudan University, Shanghai, China; 2 Ministry of Education Key Laboratory of Contemporary Anthropology, School of Life Sciences and Institutes of Biomedical Sciences, Fudan University, Shanghai, China; 3 The Fifth People’s Hospital of Wuxi, Wuxi 214073, China; 4 Department of Computational Regulatory Genomics, CAS-MPG Partner Institute for Computational Biology, Shanghai Institutes for Biological Sciences, Chinese Academy of Sciences, Shanghai, China; 5 Key Laboratory of Computational Biology, CAS-MPG Partner Institute for Computational Biology, Chinese Academy of Sciences, Shanghai, China

**Keywords:** Phylogeny, Hepatitis B virus, Recombination, Consensus tree

## Abstract

**Background:**

Hepatitis B virus (HBV) is an important infectious agent that causes widespread concern because billions of people are infected by at least 8 different HBV genotypes worldwide. However, reconstruction of the phylogenetic relationship between HBV genotypes is difficult. Specifically, the phylogenetic relationships among genotypes A, B, and C are not clear from previous studies because of the confounding effects of genotype recombination. In order to clarify the evolutionary relationships, a rigorous approach is required that can effectively explore genetic sequences with recombination.

**Result:**

In the present study, phylogenetic relationship of the HBV genotypes was reconstructed using a consensus phylogeny of phylogenetic trees of HBV genome segments. Reliability of the reconstructed phylogeny was extensively evaluated in agreements of local phylogenies of genome segments.

The reconstructed phylogenetic tree revealed that HBV genotypes B and C had a closer phylogenetic relationship than genotypes A and B or A and C. Evaluations showed the consensus method was capable to reconstruct reliable phylogenetic relationship in the presence of recombinants.

**Conclusion:**

The consensus method implemented in this study provides an alternative approach for reconstructing reliable phylogenetic relationships for viruses with possible genetic recombination. Our approach revealed the phylogenetic relationships of genotypes A, B, and C of HBV.

## Background

Hepatitis B virus (HBV), a serious global public health problem, is the 10th leading cause of death worldwide. Approximately 2 billion people worldwide are infected with this virus and about 350 million live with chronic infection. An estimated 600,000 people die each year due to acute or chronic consequences of hepatitis B [1].

There are eight well-recognized HBV genotypes, labeled A through H, each pair of which differs by at least 8% of the complete genome sequence. The distribution of the genotypes varies across geographic regions with population migration [2,3]. Type A is located mostly in Europe, South Africa, and North America; types B and C are prevalent in East Asia, Southeast Asia, and Oceania; type D is common in South Asia, the Mediterranean area, and the Middle East; type E is predominant in sub-Saharan Africa; types F, G, and H are common in the New World and are also found in some European countries, such as France and Germany. Within the 8 genotypes, HBV can be further divided into different subtypes that differ by 4% to 8% of the genome [3]. Besides the 8 well known genotypes, there are two more putative genotypes that could not be classified into those groups above, genotype I and J [4,5].

Several studies have reported controversial phylogenetic relationships among HBV genotypes, especially genotypes A, B, and C. Three reports suggest that genotypes A and C have a closer phylogenetic relationship than genotype B with A or C [4,6,7]. The above phylogenetic relationship has been brought into question, however, by the results of other studies demonstrating that genotypes B and C have a closer phylogenetic relationship than genotype A with B or C [8-10]. One study also reported that the phylogenetic relationship between genotypes A and B is much closer than that of genotype C with A or B [3]. Further, three other studies were unable to elucidate the relationship of the genotypes in detail and suggested that the three genotypes were on the same phylogenetic clade [11-13]. The ambiguity of the phylogenetic relationship of the HBV genotypes is thought to be due in part to historical recombination in the HBV genome [8,9,14]. Recent efforts have been made to detection HBV recombinants in HBV genome and provided a comprehensive picture about the distribution of recombination in HBV genome [14-16].

In order to reduce the confounding effects of recombination in the process of phylogeny reconstruction, Fares and Holmes (2001) utilized gene non-overlapping regions of the HBV genome to reconstruct the phylogeny, but the reconstructed phylogeny from their study was not consistent with the geographic prevalence of the genotypes; i.e., genotypes B and C were distributed geographically closer while they were more distant in their reconstructed phylogenetic relationship [3,6]. Therefore, it might be necessary to incorporate the whole-genome information of HBV, and it is highly unlikely that an approach that does not consider the recombination will solve the ambiguity of the phylogenetic relationship of HBV genotypes. To resolve the ambiguity, we were offered an opportunity to propose and validate effective phylogenetic methods for exploring genetic sequences with recombination.

Here, we reconstructed the phylogenetic relationship of HBV genotypes using a consensus-tree approach to integrate whole-genome information. The overall phylogeny indicated that HBV genotypes B and C have a closer phylogenetic relationship than genotype A with B or C. Multi-level evaluations implicated the reconstructed phylogenetic tree of HBV genotypes was reliable in many perspectives. We did not consider this report as a solely clarification of HBV phylogenies but rather a communication of the implemented methods. The methods implemented in this study could be an alternative choice for phylogeny reconstruction in the presence of recombinant.

## Results

### Consensus relationship of local phylogenies

**Figure 1:**
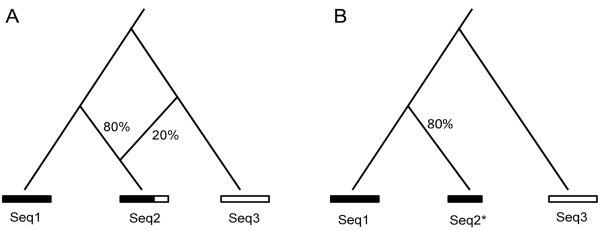
**Identifying the major phylogenetic relationship from a phylogeny with reticulation. A**. The Seq2 is a mosaic sequence in which most of its components (80%) are descendants of sequence Seq1 and the remaining components (20%) are descendants of sequence Seq3. **B**. A major phylogenetic relationship can be achieved by removing the minor relationship between Seq2 and Seq3. ‘*’ indicated this is a truncated sequence.

The phylogenetic relationship can be represented as a phylogenetic network with reticulations when recombination occurs among sequences. For three sequences with a known root, the phylogenetic relationship can be shown as a rooted triplet with reticulations (Figure 1A; a four-taxa quartet, if one of the taxa is the given out-group, then the quartet is called a rooted triplet). In this scenario, except homoplasy, formation of the reticulation can be generally explained as a consequence of recombination between sequence Seq1 and Seq3 when a recombination event is highly possible [17,18]. In the presence of recombination, sequence Seq2 could be considered as a mosaic of the Seq1 and Seq2 following the law of parsimony, i.e., Occam’s razor. We defined that the major phylogenetic relationship (shown as a rooted triplet without reticulation, Figure 1B) of the three involved sequences was the topological relationship presented by the majority of phylogenetic trees of their aligned sequence segments. In the major phylogenetic relationship, the ancestor of the mosaic is the ancestral sequence that contributed the most genetic content (80% in Figure 1) to the mosaic compared with the other sequences. When a pool of the major rooted triplets is available to present major phylogenetic relationships of all possible three-sequence combinations for multiple sequences, a consensus tree of the major rooted triplets could present the major phylogenetic relationship of all of the involved sequences.

### Tree-like phylogeny of HBV

**Figure 2:**
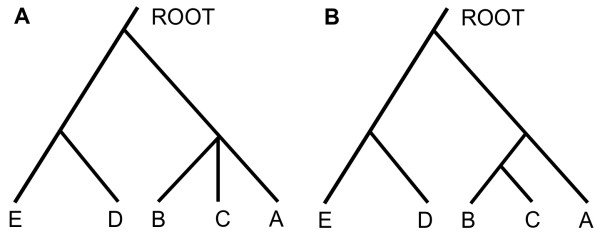
**Schematic presentation of the phylogenetic relationship of HBV genotypes A, B, and C. A**. The phylogenetic relationship of the three genotypes is ambiguous when the analyzing window was only 250 bp in size. **B**. Genotypes **B** and **C** showed a closer phylogenetic relationship when the analysis window size was at least 500 bp.

In the present study, the consensus phylogenetic relationship of the involved HBV sequences was constructed using the majority consensus of local phylogenies of all genome segments (see Methods for details). We named the phylogenetic relationship of a genome segment as the local phylogeny. When the size of all genome segments was 250 base pairs (bp), the consensus phylogenetic relationship of HBV genotypes was ambiguous such that genotypes A, B, and C appeared in the same clade of the consensus tree forming a trifurcation (Figure 2A). When the segment size was increased to 500 bp, 750 bp, 1000 bp, 1250 bp, or 1500 bp, however, the consensus topological relationship of the HBV genotypes was the same (Figure 2B). In these analyses, the B and C genotypes had a closer phylogenetic relationship than that of genotype A with B or C. The close phylogenetic relationship between genotypes B and C was strongly supported by bootstrapping evaluation (0.99, 1000 times bootstrapping). Notably, the close relationship between genotypes B and C was also supported by the worldwide geographic prevalence of the HBV genotypes and the fact that both genotypes are prevalent in East Asia [3].

### Reliability of the consensus phylogenetic relationship

A good consensus phylogenetic tree should represent the majority of phylogenetic relationships of different segments of the HBV genome for all involved sequences. To gain a thorough understanding of the reliability of our results, we evaluated the constructed consensus phylogenetic trees at both the tree and branch levels.

**Figure 3:**
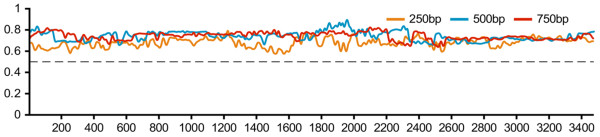
**Consistency between the consensus phylogenetic trees and corresponding local phylogenies along the HBV genome.** Consistency was measured as a percentage of the agreement between local phylogenies of different segment sizes and the corresponding consensus tree. The percentage is shown on the y-axis and the x-axis shows the coordinates of local phylogenies along the aligned HBV sequences. The dashed line indicates the 50% agreement.

At the tree level, we checked the consistencies between the constructed consensus trees and local phylogenies of sequence segments (see Methods for details). Our results indicated that the consensus trees were well-supported by the local phylogenies of sequence segments located at different coordinates (Figure 3, Additional file 1: Figure S1). The mean consistencies of different segment sizes ranged from 0.68 to 0.75 with standard deviations ranging from 0.02 to 0.05. More specifically, the mean ± standard deviation of the consistencies was 0.68 ± 0.05, 0.74 ± 0.05, 0.74 ± 0.04, 0.74 ± 0.02, 0.75 ± 0.03, and 0.72 ± 0.02 for segment sizes 250 bp, 500 bp, 750 bp, 1000 bp, 1250 bp, and 1500 bp, respectively. Further, the consistencies were sensitive to the size of the sequence segments, but there was no significant difference among different genome regions. When the segment size increased, the difference in the consistencies of different segments decreased (Additional file 1: Figure S1).

**Figure 4:**
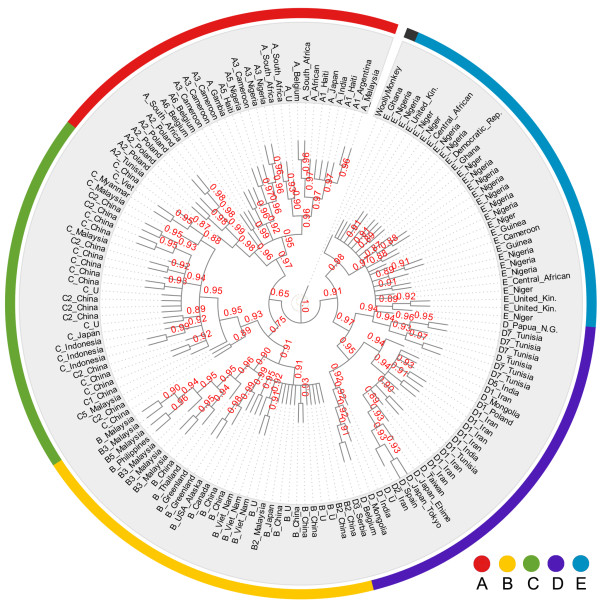
**Reliability of internal branches of the consensus phylogenetic tree.** The reliability of each internal branch is marked in red on each internal branch. Only the consensus phylogenetic tree from analyzing a 500-bp window is presented. More results for other window sizes are shown in Additional file 1: Figure S4. Accession Numbers of the HBV sequences are supplied in Additional file 1: Table S1.

At the branch level, the reliability of each internal branch of the consensus phylogenetic trees was evaluated based on the agreement of local phylogenies with the specific branch (see Methods for details). The branches of the consensus phylogenetic trees were highly reliable. Agreements of the intra-genotype branches were generally greater than 0.90 and their 95% confidence intervals (CI) were very narrow in the bootstrapping evaluation (1000 times bootstrapping, see Methods for details, Figure 4, Additional file 1: Figure S2). The high reliabilities at the branch level suggest that intra-genotype recombination has a limited impact on our reconstructed phylogenetic relationship. Reliabilities of inter-genotype branches were generally high (with agreements over 0.90), except for two branches (Figure 4, Additional file 1: Figure S2). One of the branches split genotypes B and C from the other genotypes and the other branch split genotypes A, B, and C from genotypes D and E. For example, when the segment size was 500 bp, the cluster of genotypes B and C had a relatively lower reliability of agreement (0.75 with 95% CI 0.74 - 0.76, Figure 4). In the same scenario of a 500-bp segment size, even the branch with the poorest reliability, which splits genotypes A, B, and C from the others, had agreement of 0.65 with 95% CI 0.63-0.67. Therefore, all splits of the reconstructed phylogenetic relationship of the HBV genotypes were well-supported by the majority of the local phylogenies (Figure 4, Additional file 1: Figure S2).

### Further demonstration for advantage of the consensus method

Maximum likelihood (ML) method is the most popular and comprehensive approach in studies of genetic phylogeny [19], as well as the studies of HBV evolution [6-8,12,20]. ML method builds inference on robust statistical models and searches trees for the best solution with maximum of likelihood value. Therefore, in many perspectives, the ML method performs excellent in phylogeny reconstruction [19]. To demonstrate advantage of our consensus method in the presence of recombination, we applied both our method and ML method on HBV sequences mixed with simulated genotype A/C recombinants (see Methods for details). Using datasets with moderate recombinant frequency (*f* = 0.14), the ML method reconstructed incorrect phylogenetic relationship where genotype A and C was wrongly clustered together (Additional file 1: Figure S3). By contrast, using the same synthetic datasets, our consensus method reconstructed phylogenetic relationship with correct topological pattern (Additional file 1: Figure S4). It is worth to mention that both the method produced correct phylogenies if the recombinants were rare in the simulated datasets. And further, both the methods failed to reconstruct correct phylogeny when the frequency of recombinants was very high, for example *f* = 0.60.

## Discussion

Phylogenetic trees are efficient representations of the genetic relationship of biologic sequences, although a phylogenetic network is more informative in applications involving reticulate relationships, such as those due to recombinant sequences [21]. Unfortunately, the currently available methods for reconstructing phylogenetic networks from genetic data containing recombinant sequences have very high false rates in terms of identifying the correct phylogeny [22]. In contrast, many tree-building methods have a high probability for reconstructing the correct phylogeny for sequences without recombination [23]. Phylogenies of aligned short pieces of sequences are rarely affected by recombination when recombination is not extremely frequent [24]. A consensus of the local phylogenies of short sequence fragments, therefore, can be used to represent the phylogenetic relationship of the majority of the involved HBV sequences.

Inter- and intra-genotype recombination is widely recognized as a critical factor in HBV evolution. Recombinants in sequence pool could lead to inconsistencies among local phylogenies of different fragments of the aligned sequences [17]. Recombination has thus posed a challenge to phylogenetic studies of HBV. In addition, uncertainty regarding the molecular clock also interferes with the reconstructed local phylogenies because, for short sequence fragments, mutation accumulation follows a Poisson distribution with great variance [25]. Therefore, HBV sequence fragments with an extremely small size, for example 250 bp, did not help to distinguish genotypes B and C from genotype A in this study. Both recombination and the uncertainty contribute to the inconsistency between local phylogenies. For the same reason, it is difficult to fully identify all or most recombination events or completely eliminate their impact in phylogenetic studies based on the comparison of local tree topology. In this study, the phylogenetic relationship was reconstructed without explicitly identifying instances of recombination events and the reconstructed relationship was appropriately supported by local phylogenies at both the tree and branch levels. A similar approach may facilitate the reconstruction of reliable tree-like phylogenetic relationships of viruses in future studies.

Classic phylogenetic trees often present phylogenetic relationships of aligned full-length sequences. The consensus phylogenetic relationship in this report, however, is different. This consensus phylogenetic relationship extracts information from the majority of the sequences. A small part of the sequence fragments was automatically ignored during the phylogeny reconstruction and the useful fragments may locate at different positions for different sequences. Excluded fragments of the same sequence may have the same or different genetic origins, but the origins make only minor genetic contributions to the sequences. In this way, minor ancestors of a sequence are ignored by the consensus phylogenetic tree. This method provides a natural way to extract important phylogenetic information from sequences containing recombination.

The reliability of the consensus phylogeny was evaluated by comparing the consensus phylogeny with local phylogenies of sequence segments in this study. The phylogenies were split into rooted triplets to compare the consistency of the triplets during the process. In this novel approach, more consistency indicated smaller topological differences between the phylogenies and better reliability of the consensus phylogeny. This approach overcomes an obvious limitation of the classical consensus measure. The classical measure of majority rule consensus actually showed a split consensus for all taxa without considering the number of taxa [26]. In the classical method, even a small difference in one or two branches was treated as having the same importance as a large difference between phylogenies. The evaluations in this report implemented an alternative approach in which a minor difference is distinguished from large differences. These findings provide another view of the reliability of consensus phylogenetic tree.

The phylogenetic relationships of HBV genotypes A, B, and C that were reconstructed in this study elucidated the geographic prevalence of the HBV genotypes and their phylogenetic relationship. In China and other East Asian countries, HBV carriers often have HBV genotype B or C, while most Japanese carriers have HBV genotype C. Genotype A is rare in East Asia and is found mostly in Western Europe, America, India, and Africa [3]. The global prevalence of HBV suggests that genotypes B and C have a close phylogenetic relationship. Therefore, based on the present findings, the map indicating the origin and historical dispersion of the HBV genotypes that identifies genotype A as being more closely related to genotype B or C appears to be incorrect. In fact, the controversial results about the phylogenetic relationships among these genotypes reported in previous publications [3-13] have caused confusion. Our study sheds light on the origin and historical dispersion of HBV by using a comprehensive approach to confirm that genotypes B and C are closer relatives.

The effects of recombination were eliminated in our analysis to make the result robust. Our simulation suggested that the consensus method was superior to regular ML method in the presence of recombination. The simulation also supplied clues of possible explanation for the difference between our consensus phylogenetic relationship and Shi et al.’s ML tree of HBV genotypes [16]. However, it is a limitation in our current study that this approach is not capable of indentifying historical recombination events in HBV genome. Fortunately, several publications have reported some progress in this field [14-16,27-31]. Evolutionary history of HBV genome recombination will possibly be clarified in details in future although rigorous improvements of analysis tools are necessary.

## Conclusions

Phylogenetic relationship can be reconstructed on majority of phylogenetic information of sequence segments without explicitly identifying historical recombination events. The serial phylogenetic methods proposed and employed in this study provide an effective approach for reconstructing reliable phylogenetic relationships for viruses with possible genetic recombination. In this approach, HBV genotypes B and C had a closer phylogenetic relationship than genotypes A and B or A and C.

## Methods

### Data preparation

We retrieved 3281 complete sequences of human HBV and one full-length sequence of woolly monkey HBV from the GenBank of the National Center for Biotechnology Information available on April 2011 [32]. The full sequence set comprised 320 genotype A, 387 genotype B, 836 genotype C, 383 genotype D, 221 genotype E, 72 genotype F, 15 genotype G, 19 genotype H, and 1043 unknown or uncertain genotype sequences. The genotypes assigned to the different sequences were obtained either directly from the GenBank records or from the associated publications.

All the sequences were screened to exclude entries that were related to patents, artificial mutants, and identical sequences. Further, sequences with unknown, uncertain genotype or documented recombination information were removed. The remaining sequences were aligned using the MUSCLE software with default parameters [33]. Results of the alignments were checked manually for further validation. Gaps (insertions/deletions) and all nonstandard nucleotide bases (all characters except A, C, G, T, and –) were considered as missing values in further analysis. After that, sequences with more than 20% gaps or missing data were removed. Positions of sites were identified by their relative positions to the traditional hypothetical EcoRI site in the full-genome alignments.

To achieve a fair and representative presentation for all the genotypes, we applied a multi-step procedure to remove extra sequences from the initial sequences set. In the first step, we sequentially removed sequences with high similarity to any others until all remaining sequences had a pairwise difference larger than or equal to 2.5%. After the initial cleaning, the sequence pool had 379 full-length HBV sequences (including 38 genotype A, 82 genotype B, 138 genotype C, 77 genotype D, 32 genotype E, 9 genotype F, 2 genotype G, and 3 genotype H).

From the filtered sequences, 30 sequences were randomly drawn for each of genotypes A, B, C, D, and E. Genotypes F, G, and H were not included in further analysis because the purpose of the present study was to elucidate the phylogenetic relationship of genotypes A, B, and C. Furthermore, to involving the limited sequences of genotypes F, G, and H (9 genotype F, 2 genotype G, and 3 genotype H) in the analysis may produce problematic results due to unequal number of involving sequences of each genotype. The full-length HBV sequence of woolly monkey was considered as an ancestral reference (outgroup) in this study [34]. This woolly monkey HBV sequence and the randomly selected human HBV sequences were combined together and aligned by MUSCLE with default parameter settings. To improve the data quality of the aligned sequences, GBLOCKs was used to remove aligned columns with more than half gaps or with low data quality [35,36]. In total, 105 columns (3.2%) were removed in the process. The working dataset therefore included 151 full-length sequences of HBV for further phylogenetic investigation.

### Constructing a consensus phylogenetic relationship

A sliding window approach was used in which an analyzing window moves along the aligned HBV sequences with the same step length (10 bp), but a different window size in different runs. The work of sliding window is similar with that of previous publication about recombination detection [13]. Analysis of the results from different runs with different window sizes (250 bp, 500 bp, 750 bp, 1000 bp, 1250 bp, or 1500 bp) could show how differences in window size impact phylogeny reconstruction. In each stop of the window movement, local phylogenetic trees of the aligned sequence fragments were reconstructed by Ninja software using the neighbor-joining method and Kimura 2 parameter model [37]. With the given outgroup, all the local phylogenetic trees were further split into primary rooted triplets. From each local phylogenetic tree, 551,300 ($C_{150}^{3}$, the number of combinations of any 3 sequences from the given set of 150 HBV sequences) primary rooted triplets were obtained. Because of the circular characteristic of HBV genome, the initial start of HBV sequences were concatenated at the end of the original sequences, in order to make each base have an equal coverage by the sliding window.

The primary rooted phylogenetic triplets of each window in each run were filtered to remove the minor triplets that presented two different minor phylogenetic relationships. It is worth to note here that, for every combination with 3 human HBV sequences and the root, there were three possible topologies for each window in each run and the three topologies were not compatible with each other. We took only one of the possible topologies, *i.e.* the major triplet, for further analysis. The removed triplets were less common and inconsistent with the major phylogenetic relationship presented in the same analyzing window (see Results for further details, Figure 1). The remaining rooted triplets from all the analyzing windows in the same run were then pooled together to reconstruct a consensus tree using the rooted triplet consensus method [38]. Ewing, et al. (2008) declared that the consensus method based on rooted triplets outperformed the *extended majority rule consensus strategy*[38]. We constructed consensus phylogenetic relationships of HBV genotypes in different runs separately using different window sizes.

### Evaluating the reliability of the reconstructed phylogenetic relationship

The reliability of the reconstructed phylogenetic relationship of HBV sequences can be evaluated by comparing the consensus phylogenetic relationship with phylogenetic trees of genome segments (local phylogenetic trees). Good consistency between them would indicate good reliability of the consensus phylogeny. In this study, multiple comparisons were conducted to achieve a thorough understanding of the reliability.

First the consistency of the reconstructed consensus phylogeny and local phylogenetic trees was investigated on a genome-segment level. For each genome segment, local neighbor-joining trees (involving all 151 taxa) were built using Ninja software with the aforementioned substitution model [37]. We then dissected the local neighbor-joining trees and our consensus tree-like phylogenetic relationship into rooted triplets. For phylogenies with n taxa (including an outgroup), the proportion of compatible triplets between the local tree and consensus tree could be obtained by $k/C_{n-1}^{3}$, where k is the total number of compatible triplets and $C_{n-1}^{3}$ is the number of total rooted triplets (*n* = 151 in this case). The proportions were calculated for all genome segments and then used as a measure for the agreement of reconstructed consensus phylogeny and local phylogenetic trees.

Second, the consistency of internal branches (nontrivial splits) of the consensus phylogenetic tree and local phylogenetic trees was evaluated by checking how often the nontrivial splits of the consensus tree were supported by nontrivial splits of local phylogenetic trees. For any given internal branch (with *m* children) of an *n*-taxa consensus tree (including an outgroup), the phylogenetic relationship was dissected into rooted triplets with a total number $C_{n-m-1}^{1}C_{m}^{2}$ to form a consensus rooted triplet pool. The probability that a given rooted triplet from the consensus rooted triplet pool was supported by dissected rooted triplets of local phylogenetic tree could be estimated by $y/\left(jC_{n-m-1}^{1}C_{m}^{2}\right)$, where y was the number of dissected rooted triplets of the local phylogenetic trees which shared the same phylogenetic relationships with their corresponding triplets of the consensus tree, and *j* was the total number of local neighbor-joining trees determined by the size of the sliding window and length of the moving step. The 95% CI of the estimation was obtained by a bootstrapping method in which local phylogenetic trees were randomly sampled with replacements to generate an artificial rooted triplet pool for the aforementioned evaluation.

### Performance demonstration in the presence of recombination

Synthetic data was generated by introducing simulated genotype A/C recombinants to the raw data set that was used for aforementioned investigation of HBV phylogeny. For a pair of sequences, one from each of the two genotypes, we gave the recombination probability *p*. Expected frequency of recombinants in the sequence pool of genotype A, C, and A/C recombinant could be estimated as *f* = 1 - (1 - *p*)^30^ because 30 sequences of each genotype were included in the raw data set. We considered all possible pairs of the involving sequences of genotypes A and C to simulate the occurrence of recombination between the two genotypes. When a recombination occurred between a pair of sequences with probability *p*, location of the recombinant fragment was randomly chosen on the HBV genome, and length of the recombinant fragment was determined by the empirical length distribution of recombinants from Yang et al’s study [15]. Because HBV genome is a circular molecular, we allowed recombinant fragment cover the junction of sequence end and start.

Phylogenetic relationship of the synthetic data was reconstructed by using ML method. Before the reconstruction, jModelTest2 was executed to choose the best-fit model from the 88 candidate models [39]. Since GTR + I + G model was selected as the best-fit model, a ML tree was built using the ML method implemented in PALM package [40]. The same synthetic data was also analyzed by our consensus method to produce a consensus tree. By given different probability of recombination *p,* we performed the data simulation and phylogeny reconstruction multiple times to achieve a thoughtful evaluation.

## Competing interests

The authors declare that they have no competing interests.

## Authors’ contributions

JY and WM performed the studies and the statistical analysis. HY and JL designed the study. HY, WW, and ZH participated in its coordination and helped to draft the manuscript. All authors read and approved the final manuscript.

## Supplementary Material

Additional file 1: Figure S1 Consistency of the consensus phylogenetic tree and local phylogenies along HBV genome for window size 1000 bp, 1250 bp and 1500 bp. The consistency is measured in percentage of the agreement between local phylogenies and corresponding consensus tree. The percentage is showed on y-axis. The x-axis represents coordinates of local phylogenies along HBV genome. The dashed line indicates the 50% agreement. **Figure S2**. Reliability of internal branches of the consensus phylogenetic tree. Reliability of the internal branches (nontrivial splits) of consensus phylogenetic tree is evaluated in rooted triplet prospective. The values on the branch are the median of 1000 times bootstrapping, confidence interval were not showed. The figures S2.1-S2.5 are for results of window size 250 bp, 750 bp, 1000 bp, 1250 bp, and 1500 bp, respectively. Accession Numbers of the HBV sequences were listed in **Table S1**. **Figure S3**. ML tree of a synthetic HBV dataset. With the simulated recombinants of genotype A and C, ML method failed to reconstruct correct phylogeny for synthetic data. The genotypes A and C formed a false cluster. Details of the simulated recombinants were presented in **Table S2**. **Figure S4**. Consensus tree of a synthetic dataset. Using synthetic data with simulated recombinants, our consensus method successfully restore the original phylogenetic relationship of HBV genotypes, where the genotype B and C formed the correct cluster. This figure shows the consensus phylogeny of sliding window size 500 bp. Details of the simulated recombinants were presented in **Table S2**. **Table S1.** Accession number of HBV sequences involved in phylogenetic trees. All these sequences were retrieved from the GenBank of the National Center for Biotechnology Information. **Table S2**. Details of simulated recombinants in a synthetic dataset. Click here for file

## References

[B1] Lavanchy D Hepatitis B virus epidemiology, disease burden, treatment, and current and emerging prevention and control measures J Viral Hepat 2004 11 97 107 10.1046/j.1365-2893.2003.00487.x 14996343

[B2] Jazayeri SM Alavian SM Carman WF Hepatitis B virus: origin and evolution J Viral Hepat 2010 17 229 235 10.1111/j.1365-2893.2009.01193.x 20002567

[B3] Kurbanov F Tanaka Y Mizokami M Geographical and genetic diversity of the human hepatitis B virus Hepatol Res 2010 40 14 30 10.1111/j.1872-034X.2009.00601.x 20156297

[B4] Yu H Yuan Q Ge S-X Wang H-Y Zhang Y-L Chen Q-R Zhang J Chen P-J Xia N-S Molecular and phylogenetic analyses suggest an additional hepatitis B virus genotype “I. PLoS One 2010 5 e9297 10.1371/journal.pone.0009297 20174575 PMC2824819

[B5] Tatematsu K Tanaka Y Kurbanov F Sugauchi F Mano S Maeshiro T Nakayoshi T Wakuta M Miyakawa Y Mizokami M A genetic variant of hepatitis B virus divergent from known human and ape genotypes isolated from a Japanese patient and provisionally assigned to new genotype J J Virol 2009 83 10538 10547 10.1128/JVI.00462-09 19640977 PMC2753143

[B6] Fares MA Holmes EC A revised evolutionary history of hepatitis B virus (HBV) J Mol Evol 2002 54 807 814 10.1007/s00239-001-0084-z 12029362

[B7] Bollyky PL Holmes EC Reconstructing the complex evolutionary history of hepatitis B virus J Mol Evol 1999 49 130 141 10.1007/PL00006526 10368441

[B8] Bollyky PL Rambaut A Harvey PH Holmes EC Recombination between sequences of hepatitis B virus from different genotypes J Mol Evol 1996 42 97 102 10.1007/BF02198834 8919861

[B9] Morozov V Pisareva M Groudinin M Homologous recombination between different genotypes of hepatitis B virus Gene 2000 260 55 65 10.1016/S0378-1119(00)00424-8 11137291

[B10] Takahashi K Brotman B Usuda S Mishiro S Prince AM Full-genome sequence analyses of hepatitis B virus (HBV) strains recovered from chimpanzees infected in the wild: implications for an origin of HBV Virology 2000 267 58 64 10.1006/viro.1999.0102 10648183

[B11] Vieth S Manegold C Drosten C Nippraschk T Günther S Sequence and phylogenetic analysis of hepatitis B virus genotype G isolated in Germany Virus Genes 2002 24 153 156 10.1023/A:1014572600432 12018706

[B12] Kidd-Ljunggren K Miyakawa Y Kidd AH Genetic variability in hepatitis B viruses J Gen Virol 2002 83 1267 1280 12029141 10.1099/0022-1317-83-6-1267

[B13] Alestig E Hannoun C Horal P Lindh M Phylogenetic origin of hepatitis B virus strains with precore C-1858 variant J Clin Microbiol 2001 39 3200 3203 10.1128/JCM.39.9.3200-3203.2001 11526151 PMC88319

[B14] Simmonds P Midgley S Recombination in the genesis and evolution of hepatitis B virus genotypes J Virol 2005 79 15467 15476 10.1128/JVI.79.24.15467-15476.2005 16306618 PMC1316029

[B15] Yang J Xing K Deng R Wang J Wang X Identification of Hepatitis B virus putative intergenotype recombinants by using fragment typing J Gen Virol 2006 87 2203 10.1099/vir.0.81752-0 16847116

[B16] Shi W Carr MJ Dunford L Zhu C Hall WW Higgins DG Identification of novel inter-genotypic recombinants of human hepatitis B viruses by large-scale phylogenetic analysis Virology 2012 427 51 59 10.1016/j.virol.2012.01.030 22374235

[B17] Posada C Intraspecific gene genealogies: trees grafting into networks Trends Ecol Evol (Amst.) 2001 16 37 45 10.1016/S0169-5347(00)02026-7 11146143

[B18] Makarenkov V Legendre P From a phylogenetic tree to a reticulated network J Comput Biol 2004 11 195 212 10.1089/106652704773416966 15072696

[B19] Whelan S Liò P Goldman N Molecular phylogenetics: state-of-the-art methods for looking into the past Trends Genet 2001 17 262 272 10.1016/S0168-9525(01)02272-7 11335036

[B20] Yang Z Lauder IJ Lin HJ Molecular evolution of the hepatitis B virus genome J Mol Evol 1995 41 587 596 7490773 10.1007/BF00175817

[B21] Huson DH Bryant D Application of phylogenetic networks in evolutionary studies Mol Biol Evol 2006 23 254 267 16221896 10.1093/molbev/msj030

[B22] Woolley SM Posada D Crandall KA A comparison of phylogenetic network methods using computer simulation PLoS One 2008 3 e1913 10.1371/journal.pone.0001913 18398452 PMC2275308

[B23] Mihaescu R Levy D Pachter L Why neighbor-joining works Algorithmica 2009 54 1 24 10.1007/s00453-007-9116-4

[B24] Martin DP Williamson C Posada D RDP2: recombination detection and analysis from sequence alignments Bioinformatics 2005 21 260 262 10.1093/bioinformatics/bth490 15377507

[B25] Duffy S Shackelton LA Holmes EC Rates of evolutionary change in viruses: patterns and determinants Nat Rev Genet 2008 9 267 276 18319742 10.1038/nrg2323

[B26] Bryant D A classification of consensus methods for phylogenetics DIMACS series in discrete mathematics and theoretical computer science 2003 61 163 184

[B27] Lyons S Sharp C LeBreton M Djoko CF Kiyang JA Lankester F Bibila TG Tamoufé U Fair J Wolfe ND Simmonds P Species association of hepatitis B virus (HBV) in non-human apes; evidence for recombination between gorilla and chimpanzee variants PLoS One 2012 7 e33430 10.1371/journal.pone.0033430 22432021 PMC3303819

[B28] Trinks J Cuestas ML Tanaka Y Mathet VL Minassian ML Rivero CW Benetucci JA Gímenez ED Segura M Bobillo MC Corach D Ghiringhelli PD Sánchez DO Avila MM Peralta LAM Kurbanov F Weissenbacher MC Simmonds P Mizokami M Oubiña JR Two simultaneous hepatitis B virus epidemics among injecting drug users and men who have sex with men in Buenos Aires, Argentina: characterization of the first D/A recombinant from the American continent J Viral Hepat 2008 15 827 838 18507755 10.1111/j.1365-2893.2008.00997.x

[B29] Fang Z-L Hué S Sabin CA Li G-J Yang J-Y Chen Q-Y Fang K-X Huang J Wang X-Y Harrison TJ A complex hepatitis B virus (X/C) recombinant is common in Long An county, Guangxi and may have originated in southern China J Gen Virol 2011 92 402 411 10.1099/vir.0.026666-0 20965984 PMC3081081

[B30] Zhou B Xiao L Wang Z Chang ET Chen J Hou J Geographical and ethnic distribution of the HBV C/D recombinant on the Qinghai-Tibet Plateau PLoS One 2011 6 e18708 10.1371/journal.pone.0018708 21494570 PMC3073994

[B31] Zhou B Wang Z Yang J Sun J Li H Tanaka Y Mizokami M Hou J Novel evidence of HBV recombination in family cluster infections in western China PLoS One 2012 7 e38241 10.1371/journal.pone.0038241 22675528 PMC3366946

[B32] Benson DA Karsch-Mizrachi I Lipman DJ Ostell J Sayers EW GenBank Nucleic Acids Res 2011 39 D32 D37 10.1093/nar/gkq1079 21071399 PMC3013681

[B33] Edgar RC MUSCLE: a multiple sequence alignment method with reduced time and space complexity BMC Bioinforma 2004 5 113 10.1186/1471-2105-5-113 PMC517706 15318951

[B34] Arauz-Ruiz P Norder H Robertson BH Magnius LO Genotype H: a new Amerindian genotype of hepatitis B virus revealed in Central America J Gen Virol 2002 83 2059 2073 12124470 10.1099/0022-1317-83-8-2059

[B35] Castresana J Selection of conserved blocks from multiple alignments for their use in phylogenetic analysis Mol Biol Evol 2000 17 540 552 10.1093/oxfordjournals.molbev.a026334 10742046

[B36] Talavera G Castresana J Improvement of phylogenies after removing divergent and ambiguously aligned blocks from protein sequence alignments Syst Biol 2007 56 564 577 10.1080/10635150701472164 17654362

[B37] Wheeler T Salzberg SL, Warnow T Large-scale neighbor-joining with ninja Proceedings of the 9th International Workshop on Algorithms in Bioinformatics: 12-13 September 2009; Philadelphia 2009 Berlin Heidelberg: Springer 375 389

[B38] Ewing GB Ebersberger I Schmidt HA Von Haeseler A Rooted triple consensus and anomalous gene trees BMC Evol Biol 2008 8 118 10.1186/1471-2148-8-118 18439266 PMC2409437

[B39] Darriba D Taboada GL Doallo R Posada D jModelTest 2: more models, new heuristics and parallel computing Nat Methods 2012 9 772 22847109 10.1038/nmeth.2109 PMC4594756

[B40] Guindon S Gascuel O A simple, fast, and accurate algorithm to estimate large phylogenies by maximum likelihood Systematic Biology 2003 52 696 704 10.1080/10635150390235520 14530136

