# Supplementary material for: Resolving ambiguity in the phylogenetic relationship of genotypes A, B, and C of hepatitis B virus
Source: BMC Evol Biol. 2013 Jun 11;13:120. doi: 10.1186/1471-2148-13-120 (PMC3682936; doi:10.1186/1471-2148-13-120)
Supplement: Additional file 1: Figure S1 — Consistency of the consensus phylogenetic tree and local phylogenies along HBV genome for window size 1000 bp, 1250 bp and 1500 bp. The consistency is measured in percentage of the agreement between local phylogenies and corresponding consensus tree. The percentage is showed on y-axis. The x-axis represents coordinates of local phylogenies along HBV genome. The dashed line indicates the 50% agreement. Figure S2. Reliability of internal branches of the consensus phylogenetic tree. Reliability of the internal branches (nontrivial splits) of consensus phylogenetic tree is evaluated in rooted triplet prospective. The values on the branch are the median of 1000 times bootstrapping, confidence interval were not showed. The figures S2.1-S2.5 are for results of window size 250 bp, 750 bp, 1000 bp, 1250 bp, and 1500 bp, respectively. Accession Numbers of the HBV sequences were listed in Table S1. Figure S3. ML tree of a synthetic HBV dataset. With the simulated recombinants of genotype A and C, ML method failed to reconstruct correct phylogeny for synthetic data. The genotypes A and C formed a false cluster. Details of the simulated recombinants were presented in Table S2. Figure S4. Consensus tree of a synthetic dataset. Using synthetic data with simulated recombinants, our consensus method successfully restore the original phylogenetic relationship of HBV genotypes, where the genotype B and C formed the correct cluster. This figure shows the consensus phylogeny of sliding window size 500 bp. Details of the simulated recombinants were presented in Table S2. Table S1. Accession number of HBV sequences involved in phylogenetic trees. All these sequences were retrieved from the GenBank of the National Center for Biotechnology Information. Table S2. Details of simulated recombinants in a synthetic dataset. [file 1471-2148-13-120-S1.doc]

**Legends of supplementary**

**Figure S1. Consistency of the consensus phylogenetic tree and local phylogenies along HBV genome for window size 1000bp, 1250 bp and 1500 bp.** The consistency is measured in percentage of the agreement between local phylogenies and corresponding consensus tree. The percentage is showed on y-axis. The x-axis represents coordinates of local phylogenies along HBV genome. The dashed line indicates the 50% agreement.

**Figure S2. Reliability of internal branches of the consensus phylogenetic tree.** Reliability of the internal branches (nontrivial splits) of consensus phylogenetic tree is evaluated in rooted triplet prospective. The values on the branch are the median of 1000 times bootstrapping, confidence interval were not showed. The figures S2.1-S2.5 are for results of window size 250 bp, 750 bp, 1000 bp, 1250 bp, and 1500 bp, respectively. Accession Numbers of the HBV sequences were listed in Table S1.

**Figure S3. ML tree of a synthetic HBV dataset.** With the simulated recombinants of genotype A and C, ML method failed to reconstruct correct phylogeny for synthetic data. The genotypes A and C formed a false cluster. Details of the simulated recombinants were presented in Table S2.

**Figure S4. Consensus tree of a synthetic dataset.** Using synthetic data with simulated recombinants, our consensus method successfully restore the original phylogenetic relationship of HBV genotypes, where the genotype B and C formed the correct cluster. This figure shows the consensus phylogeny of sliding window size 500bp. Details of the simulated recombinants were presented in Table S2.

**Figure S1**


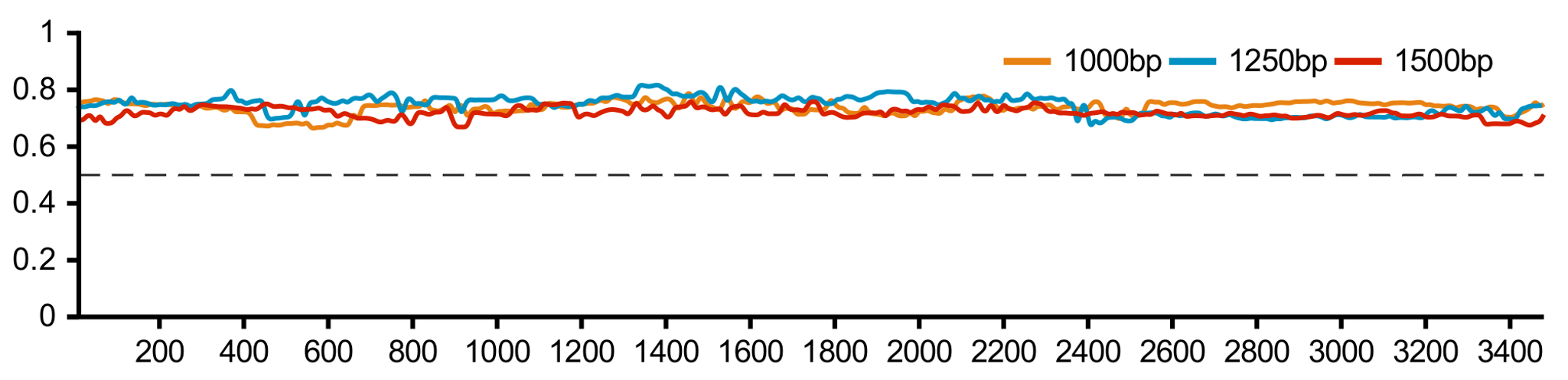


**Figure S2.1**

**
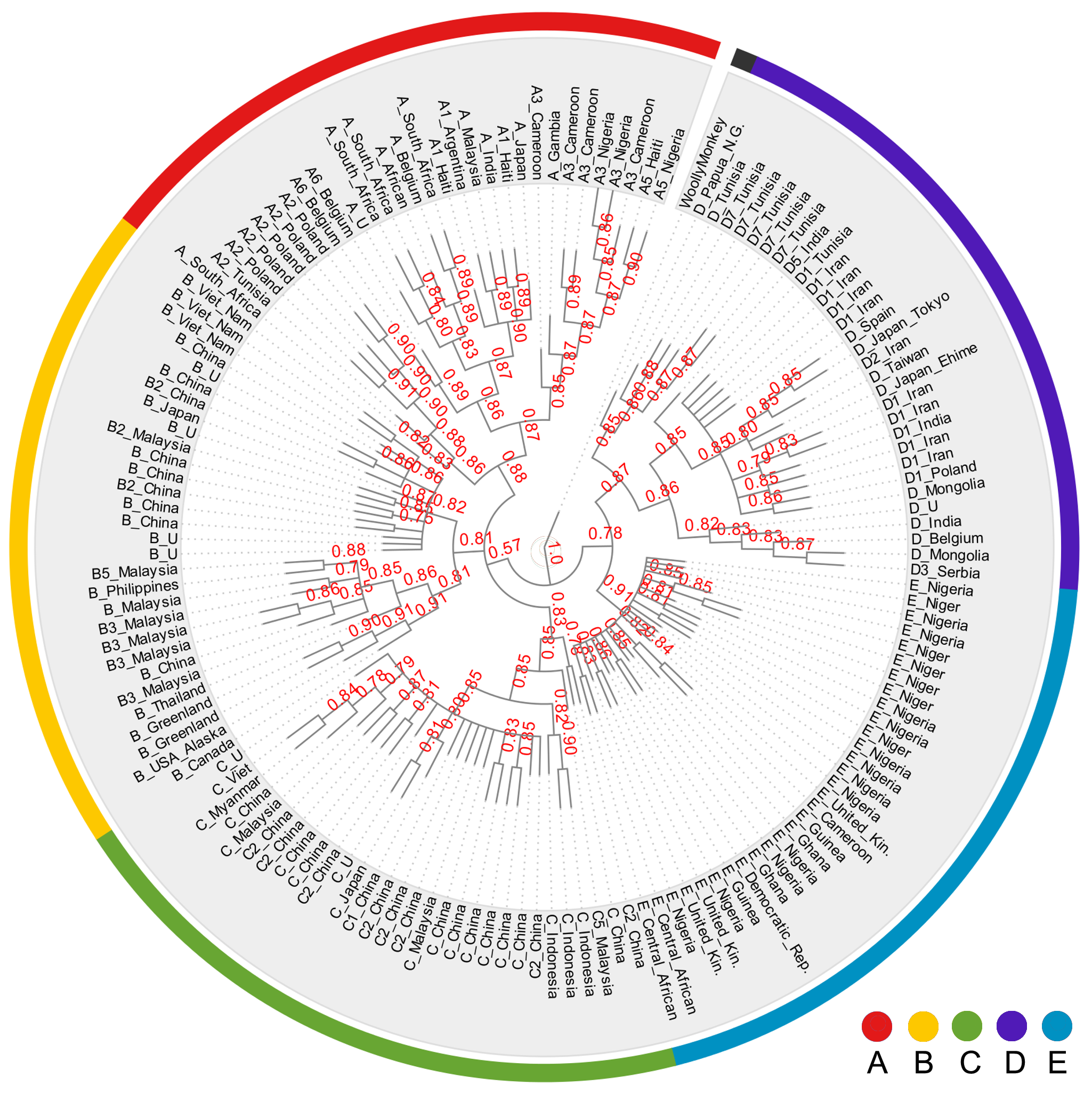
**

**Figure S2.2**

**
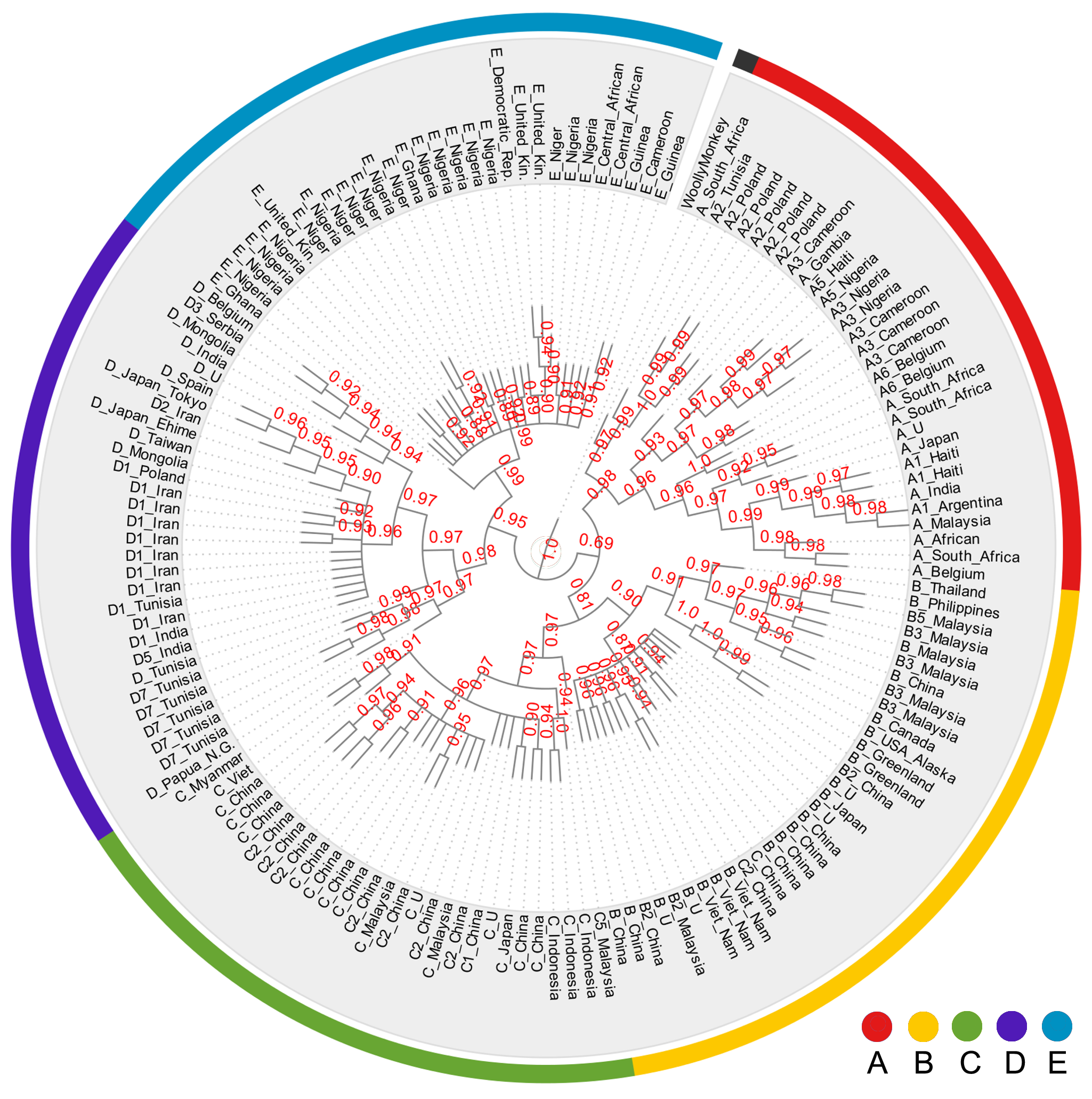
**

**Figure S2.3**

**
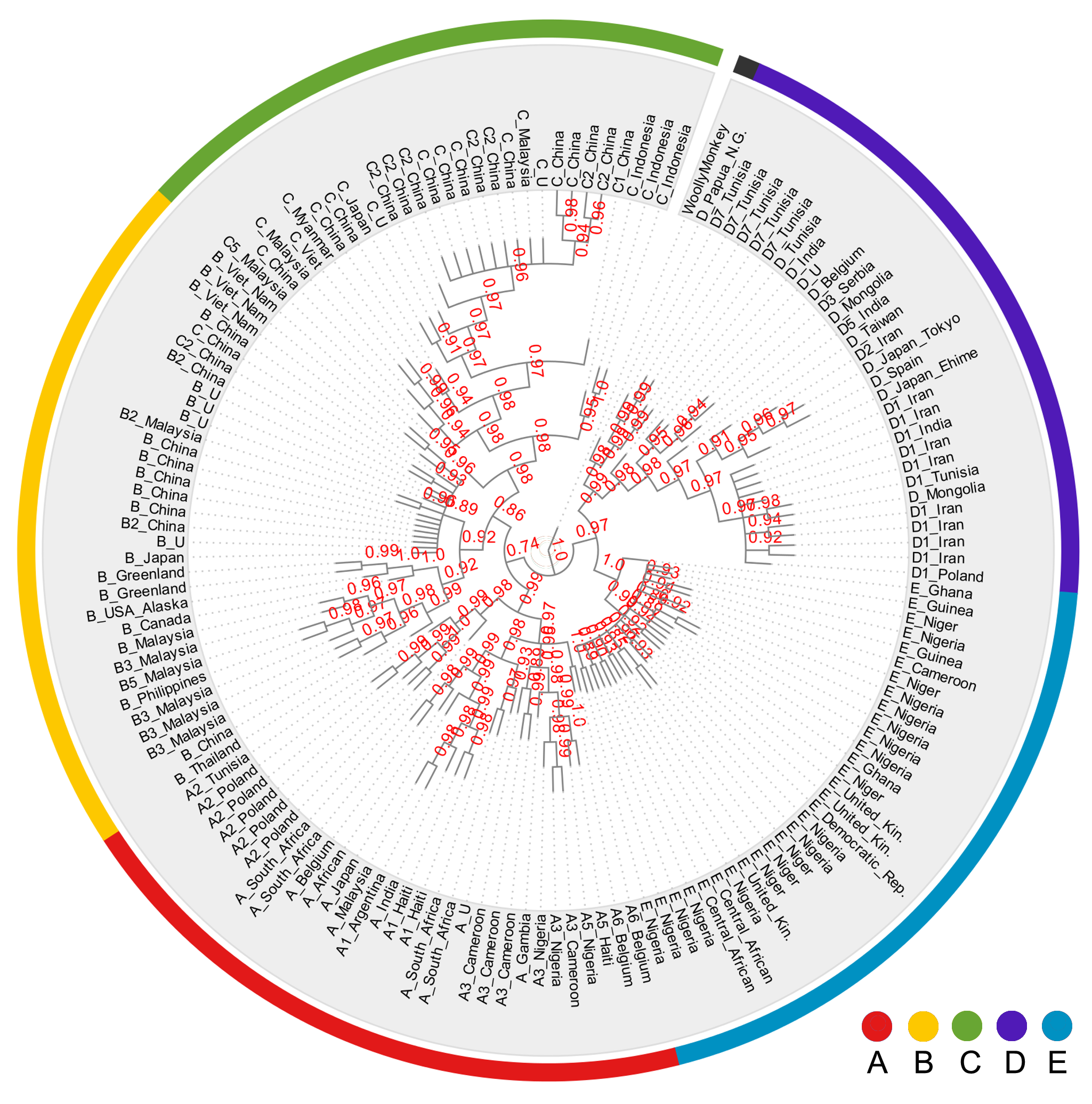
**

**Figure S2.4**

**
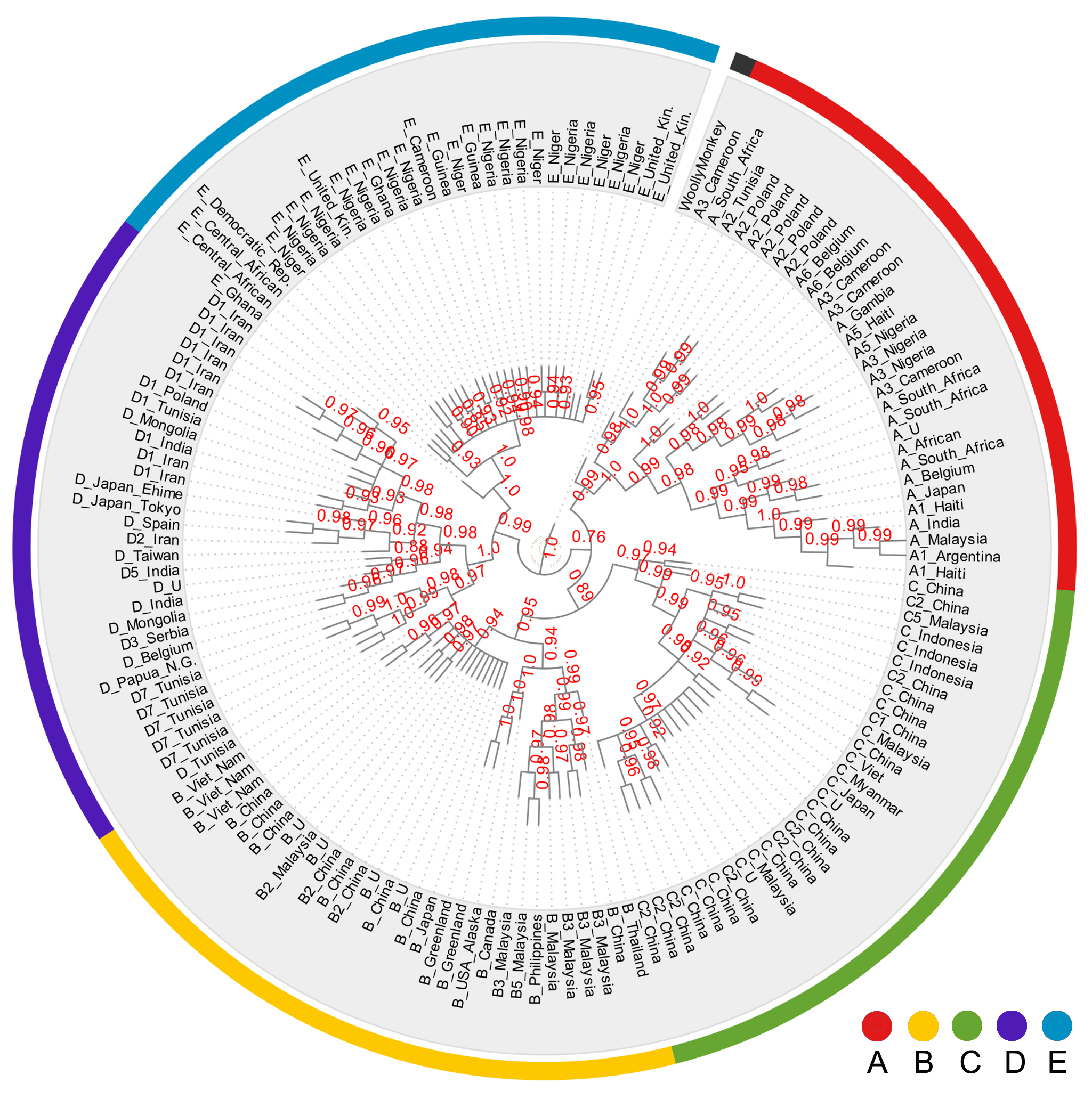
**

**Figure S2.5**

**
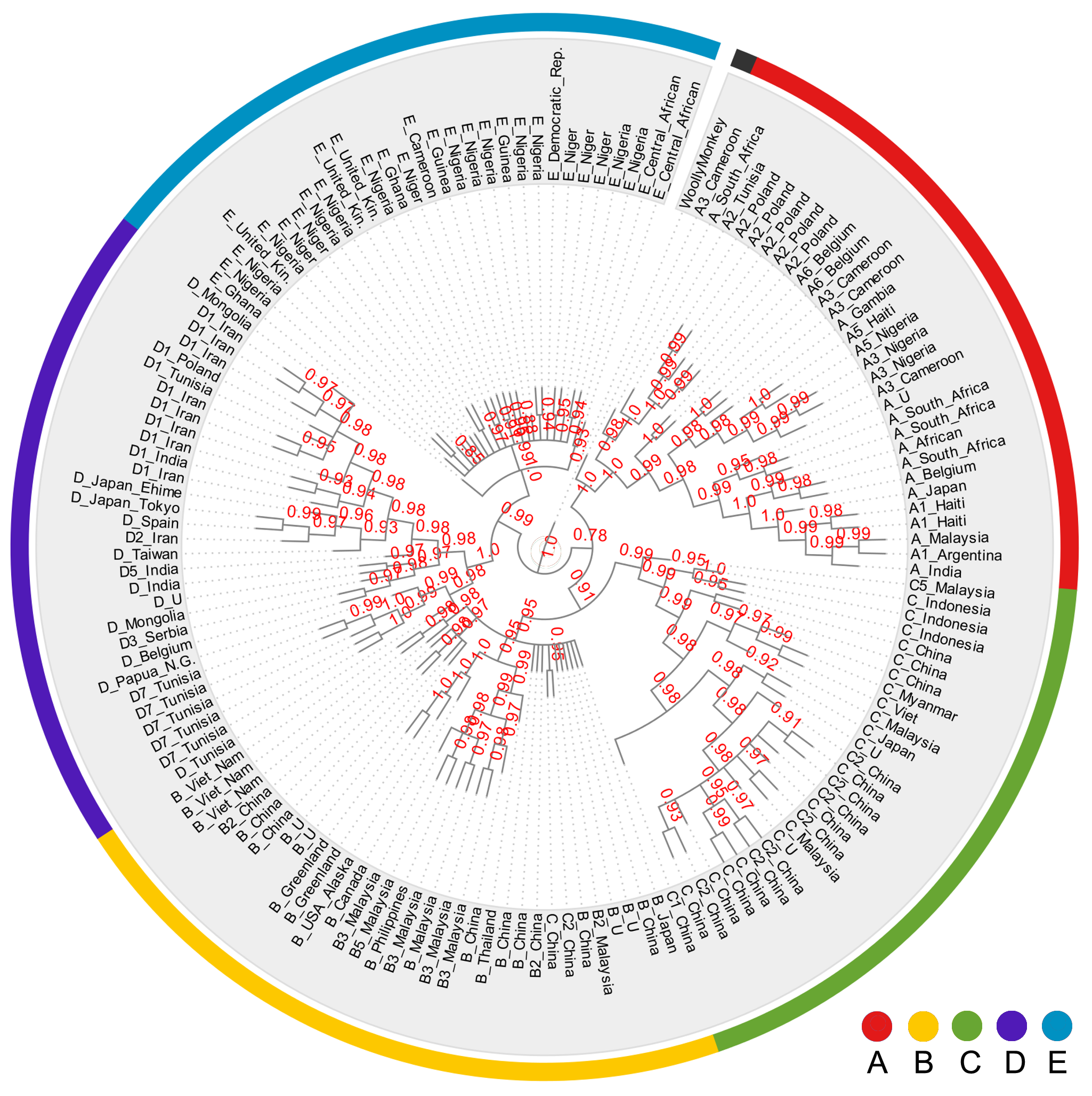
**

**Figure S3**

**
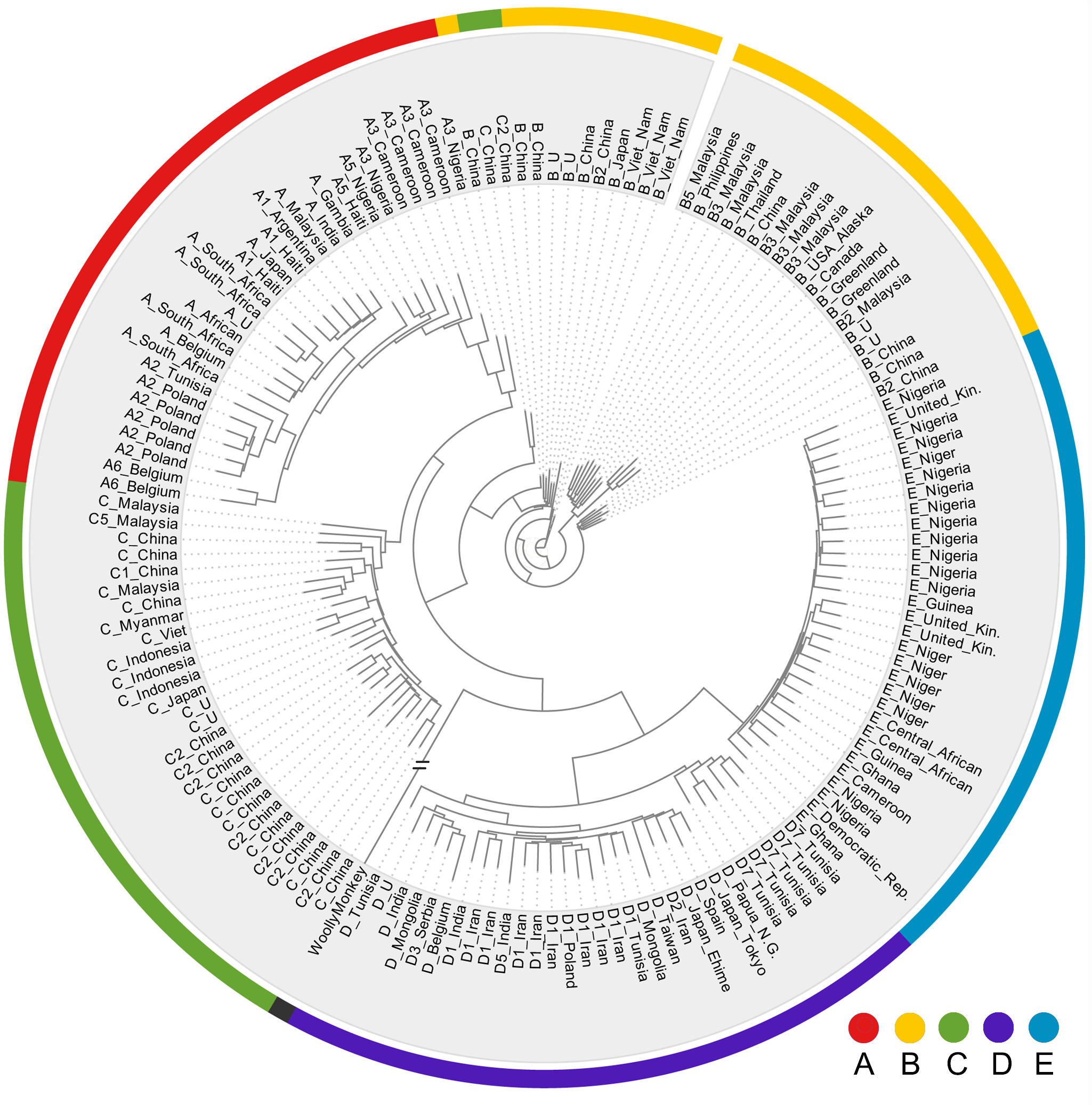
**

**Figure S4**

**
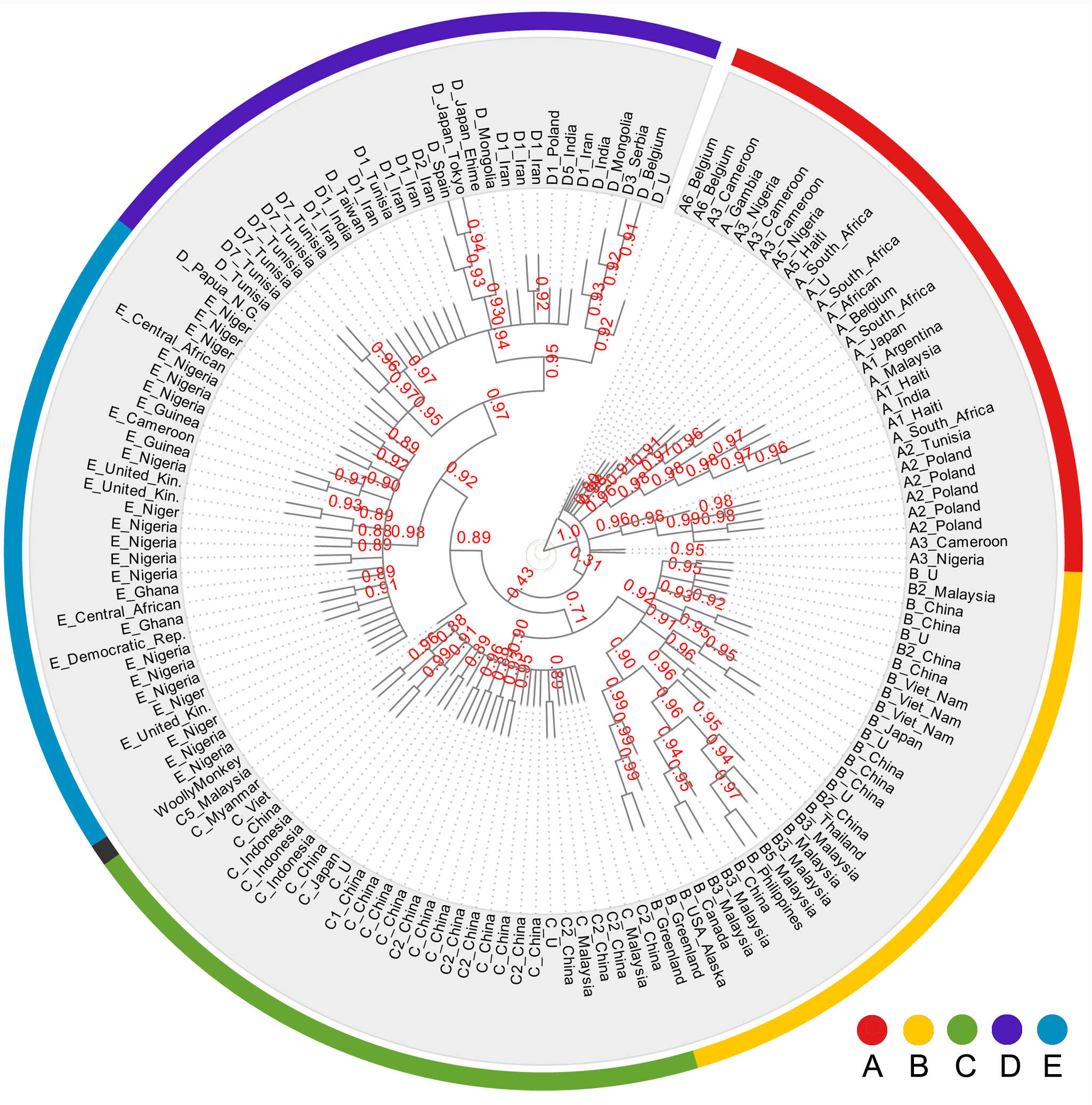
**

**Table S1. Accession number of HBV sequences involved in phylogenetic trees.** All these sequences were retrieved from the GenBank of the National Center for Biotechnology Information.

| **Accession Number** |
| --- |
| **AF046996.1_WoollyMonkey** |
| **HM363611.1_E_Nigeria** |
| **EU239220.1_E_Ghana** |
| **AB219534.1_E_United_Kin.** |
| **HM363610.1_E_Nigeria** |
| **HM363606.1_E_Nigeria** |
| **HM363604.1_E_Nigeria** |
| **FN594751.1_E_Niger** |
| **HM363608.1_E_Nigeria** |
| **AB106564.1_E_Ghana** |
| **HM363602.1_E_Nigeria** |
| **GQ161825.1_E_Guinea** |
| **HM363609.1_E_Nigeria** |
| **AB219529.1_E_United_Kin.** |
| **AB219533.1_E_United_Kin.** |
| **HM363605.1_E_Nigeria** |
| **HM363607.1_E_Nigeria** |
| **HM363600.1_E_Nigeria** |
| **HM363565.1_E_Nigeria** |
| **FN594762.1_E_Niger** |
| **HM363603.1_E_Nigeria** |
| **HM363599.1_E_Nigeria** |
| **AY738144.1_E_Democratic_Rep.** |
| **FN594761.1_E_Niger** |
| **AM494692.1_E_Central_African** |
| **AM494691.1_E_Central_African** |
| **FN594757.1_E_Niger** |
| **AB194948.1_E_Cameroon** |
| **GQ161823.1_E_Guinea** |
| **FN594765.1_E_Niger** |
| **FN594753.1_E_Niger** |
| **AB555500.1_D_Taiwan** |
| **FJ904425.1_D7_Tunisia** |
| **FJ904400.1_D7_Tunisia** |
| **FJ904417.1_D7_Tunisia** |
| **FJ904439.1_D7_Tunisia** |
| **FJ904442.1_D7_Tunisia** |
| **FJ349221.1_D_Belgium** |
| **GQ205380.1_D5_India** |
| **AB033559.1_D_Papua_N.G.** |
| **GU456678.1_D1_Iran** |
| **AB188243.2_D_U** |
| **GQ183486.1_D_India** |
| **AB270537.1_D_Mongolia** |
| **HQ236015.1_D3_Serbia** |
| **AB210818.1_D_Japan_Ehime** |
| **AY090452.1_D_Spain** |
| **AB210820.1_D_Japan_Tokyo** |
| **GU456635.1_D2_Iran** |
| **GU456639.1_D1_Iran** |
| **GU456679.1_D1_Iran** |
| **GU456665.1_D1_Iran** |
| **GU456684.1_D1_Iran** |
| **GU456657.1_D1_Iran** |
| **GU456654.1_D1_Iran** |
| **GQ477458.1_D1_Poland** |
| **FJ904426.1_D1_Tunisia** |
| **AB270550.1_D_Mongolia** |
| **FJ904422.1_D_Tunisia** |
| **GU456643.1_D1_Iran** |
| **DQ315778.1_D1_India** |
| **GQ331048.1_A6_Belgium** |
| **GQ331046.1_A6_Belgium** |
| **AY934763.1_A_Gambia** |
| **AB194949.1_A3_Cameroon** |
| **AB194950.1_A3_Cameroon** |
| **AM180624.1_A3_Cameroon** |
| **FJ692595.1_A5_Haiti** |
| **FJ692556.1_A5_Nigeria** |
| **AB194951.1_A3_Cameroon** |
| **HM363612.1_A3_Nigeria** |
| **HM363613.1_A3_Nigeria** |
| **EU366129.2_A1_Argentina** |
| **FJ692558.1_A1_Haiti** |
| **FJ692589.1_A1_Haiti** |
| **AB453986.1_A_Japan** |
| **HM011485.1_A_Malaysia** |
| **AY373428.1_A_India** |
| **GU563547.1_A_Belgium** |
| **AF297625.1_A_African** |
| **AY233281.1_A_South_Africa** |
| **AY233290.1_A_South_Africa** |
| **AF297621.1_A_U** |
| **U87742.3_A_South_Africa** |
| **U87746.3_A_South_Africa** |
| **FJ904434.1_A2_Tunisia** |
| **GQ477481.1_A2_Poland** |
| **GQ477502.1_A2_Poland** |
| **GQ477473.1_A2_Poland** |
| **GQ477474.1_A2_Poland** |
| **GQ477493.1_A2_Poland** |
| **GQ924620.1_C5_Malaysia** |
| **AB560662.1_C_Indonesia** |
| **AB554025.1_C_Indonesia** |
| **AP011108.1_C_Indonesia** |
| **AB115417.1_C_Japan** |
| **FJ562300.1_C1_China** |
| **FJ386626.1_C2_China** |
| **JF436923.1_C_China** |
| **EU547558.1_C_Malaysia** |
| **AB112065.1_C_Viet** |
| **AB112408.1_C_Myanmar** |
| **EU939624.1_C_China** |
| **AY206386.1_C_U** |
| **FJ386651.1_C2_China** |
| **FJ386628.1_C2_China** |
| **FJ562250.1_C_China** |
| **FJ562267.1_C_China** |
| **EU939596.1_C_China** |
| **FJ562320.1_C2_China** |
| **EU787444.1_C2_China** |
| **EU939653.1_C_China** |
| **AB042285.1_C_U** |
| **FJ787487.1_C2_China** |
| **EU939548.1_C_China** |
| **FJ562255.1_C_China** |
| **FJ899783.1_C2_China** |
| **HM011481.1_C_Malaysia** |
| **EU939537.1_C_China** |
| **DQ463795.1_B_Canada** |
| **AB287316.1_B_USA_Alaska** |
| **AB287321.1_B_Greenland** |
| **AB287320.1_B_Greenland** |
| **EU939629.1_B_China** |
| **AY800392.1_B_China** |
| **HM011487.1_B_Malaysia** |
| **DQ361535.1_B_Thailand** |
| **GQ924635.1_B3_Malaysia** |
| **GQ924656.1_B3_Malaysia** |
| **AB241117.1_B_Philippines** |
| **GQ924640.1_B5_Malaysia** |
| **GQ924637.1_B3_Malaysia** |
| **GQ924617.1_B3_Malaysia** |
| **AB287327.1_B_Japan** |
| **EU939623.1_C_China** |
| **FJ562328.1_C2_China** |
| **AY206383.1_B_U** |
| **JF436921.1_B_China** |
| **EU939627.1_B_China** |
| **FJ787477.1_B2_China** |
| **AY217355.1_B_U** |
| **EU939636.1_B_China** |
| **EU939635.1_B_China** |
| **AY206390.1_B_U** |
| **AY206380.1_B_U** |
| **GQ924659.1_B2_Malaysia** |
| **EU158263.1_B_China** |
| **FJ562311.1_B2_China** |
| **DQ993687.1_B_Viet_Nam** |
| **DQ993682.1_B_Viet_Nam** |
| **DQ993681.1_B_Viet_Nam** |

**Table S2. Details of simulated recombinants in a synthetic dataset.**

| Genotype A | Genotype C | Segment Length | Start | End |
| --- | --- | --- | --- | --- |
| GQ331046.1_A6_Belgium | AB042285.1_C_U | 331 | 593 | 924 |
| AB194949.1_A3_Cameroon | HM011481.1_C_Malaysia | 347 | 414 | 761 |
| AB194950.1_A3_Cameroon | EU939653.1_C_China | 244 | 1979 | 2223 |
| AB194950.1_A3_Cameroon | FJ787487.1_C2_China | 556 | 1695 | 2251 |
| HM363613.1_A3_Nigeria | EU787444.1_C2_China | 599 | 1788 | 2387 |
| HM363613.1_A3_Nigeria | HM011481.1_C_Malaysia | 704 | 2314 | 3018 |
